# The diagnostic value of ultrasound shear wave elastography combined with fecal biomarkers testing in children with inflammatory bowel disease

**DOI:** 10.12669/pjms.41.8.12421

**Published:** 2025-08

**Authors:** Li Yang, Bailing Liu, Huan He, Qi Zhang, Qian Zhang, Zhan Zhang

**Affiliations:** 1 Li Yang Department of Ultrasound, Xi’an Children’s Hospital, Xi’an, Shaanxi Province 710003, P.R. China; 2 Bailing Liu Department of Ultrasound, Xi’an Children’s Hospital, Xi’an, Shaanxi Province 710003, P.R. China; 3 Huan He Department of Ultrasound, Xi’an Children’s Hospital, Xi’an, Shaanxi Province 710003, P.R. China; 4 Qi Zhang Department of Ultrasound, Xi’an Children’s Hospital, Xi’an, Shaanxi Province 710003, P.R. China; 5 Qian Zhang Department of Ultrasound, Xi’an Children’s Hospital, Xi’an, Shaanxi Province 710003, P.R. China; 6 Zhan Zhang Department of Ultrasound, Xi’an Children’s Hospital, Xi’an, Shaanxi Province 710003, P.R. China

**Keywords:** Diagnostic, Shear wave elastography, Fecal biomarkers, Children, Inflammatory bowel disease

## Abstract

**Objective:**

To investigate the diagnostic value of ultrasound shear wave elastography (SWE) in combination with fecal biomarkers in children with inflammatory bowel disease (IBD).

**Methods:**

This retrospective cohort analysis included clinical data from children who underwent SWE and fecal biomarker testing at Xi’an Children’s Hospital from January 2022 to December 2023 for confirmed or suspected IBD. In this study, 120 children diagnosed with IBD were matched with a non-IBD cohort in a 1:1 ratio. Fecal biomarkers and SWE parameters of both groups were analyzed.

**Results:**

The levels of fecal calprotectin (FC), fecal lactoferrin (FL), Limberg score, elastic modulus standard deviation (SD), and Young’s modulus values in the IBD group were higher than those in the non-IBD group (P < 0.05). Spearman’s test confirmed a significant positive correlation between the severity of IBD and the levels of FC, FL, Limberg score, elastic modulus SD value, and Young’s modulus value (P < 0.05). Logistic regression analysis identified high FC, FL, elastic modulus SD values, and Young’s modulus values as significant risk factors for a poor prognosis in IBD (P < 0.05). The receiver operating characteristic (ROC) curve showed that the combined value of the above four indicators for predicting poor prognosis in children with IBD is higher than that of each individual indicator.

**Conclusions:**

SWE combined with fecal biomarkers testing has high diagnostic and prognostic value in children with IBD.

## INTRODUCTION

Inflammatory bowel disease (IBD) is a chronic, non-specific intestinal disorder that encompasses both ulcerative colitis (UC) and Crohn’s disease (CD).^1^ The incidence of pediatric IBD has increased significantly in recent years, and it has been shown that the highest annual pediatric incidences of IBD were 23/100000 person-years in Europe, 15.2/100000 in North America, and 11.4/100000 in Asia/the Middle East and Oceania.^2^,^3^ Because of the chronic recurrent nature of IBD, children with the disease will require multiple follow-up examinations throughout their lives. Currently, histopathological and endoscopic examinations are commonly used in clinical practice to diagnose and evaluate IBD.^1^,^2^,^4^ However, repeated endoscopic examinations can increase the physical, mental, and economic burden on patients, and endoscopic examinations may cause complications such as bleeding and perforation.^4^,^5^ Other methods, such as computer tomography (CT) or magnetic resonance imaging (MRI), are associated with radiation exposure risks and higher costs, respectively.^6^ Therefore, ultrasound has become increasingly valued in the diagnosis and follow-up of children with IBD due to its high sensitivity and specificity, lack of radiation, simplicity, and cost-effectiveness.^7^,^8^

Real-time shear wave elastography (SWE) is a novel ultrasound elastography technique that can clearly identify lesion characteristics and depth range, as well as quantitatively evaluate tissue stiffness.^9^ It has been increasingly used in IBD, especially for differentiating intestinal fibrosis in CD.^9^ Fecal biomarker testing is also an important diagnostic method for IBD.^10^,^11^ Research showed that IBD patients exhibit varying degrees of elevated fecal calprotectin (FC) and fecal lactoferrin (FL) levels, and their disease status can be determined by measuring these levels with clear advantages of simple non-invasive procedure and low cost.^10^,^11^ However, there are few reports on the application of SWE combined with fecal biomarker testing in IBD. This study aimed to evaluate the diagnostic and follow-up value of SWE and fecal biomarker testing in pediatric patients with IBD.

## METHODS

This retrospective cohort analysis included data from children who underwent fecal biomarker and SWE tests at Xi’an Children’s Hospital between January 2022 and December 2023 for confirmed or suspected IBD. In this study, 120 children diagnosed with IBD were matched with a non-IBD cohort in a 1:1 ratio. The matching criteria were age and body mass index (BMI).

### Ethical Approval:

The ethics committee of our hospital approved the study with the number 20250110-07, date: January 13, 2025.

### Inclusion criteria:

- Children who underwent fecal biomarker testing and SWE for confirmed or suspected IBD.
- Diagnosis based on comprehensive clinical manifestations through histopathological examination and endoscopic examination.
- Age range from 2 to 12 years old;
- Complete clinical data.

### Exclusion criteria:

- Individuals with upper gastrointestinal ulcers/erosions or acute infectious gastrointestinal diseases.
- Individuals with other digestive system diseases.
- Individuals with respiratory and other circulatory system diseases.
- Individuals with history of abdominal surgery over the past year.

### Fecal biomarker detection:

Patients were required to collect fecal samples in the morning within five days and store them at room temperature before testing. FC levels were measured using a human calprotectin-linked immunosorbent assay kit (Cell Sciences, Canton, MA, USA). FL levels were measured using a high-throughput discrete clinical chemistry analyzer (Hemo Techt NS Plus C, Alfresa Pharma Corp.) and colloidal gold coagulation reagent (Auto Lf Plus, Alfreda Pharma Corp, Osaka, Japan).

### SWE

### Two-dimensional ultrasound:

Patients were instructed to fast for 6-8 hours prior to the examination. The child was guided to lie flat. The ultrasound was performed using the Hitachi ALOCK ultrasound diagnostic instrument, and a high-frequency ultrasound probe (C8-5) was used to scan the entire abdomen, starting from the sigmoid colon and proceeding counterclockwise. Long axis and short axis scanning were implemented in segmented form. After identifying the positive area, the high-frequency linear array probe ML6-15 was used for further examination. The full thickness of the intestinal wall was measured in the sigmoid colon, mid descending colon, colon spleen, colon liver, mid ascending colon, ileocecal region, lower ileum, and positive area (from the high echogenicity line between the intestinal lumen and mucosal layer to the high echogenicity line between the intrinsic muscle layer and serosa). If multiple intestinal segments were involved, the maximum wall thickness was taken as the final result.

### Doppler ultrasound:

The thickest part of the intestinal wall in the sigmoid colon, mid descending colon, colon spleen, colon liver, mid ascending colon, ileocecal, lower ileum, and positive areas were explored. The high-frequency linear array probe ML6-15 was used, the area of the color sampling frame was adjusted, flicker artifacts reduced, the energy gain was adjusted to ensure that the sampling frame was entirely covered by color, and then gradually shrunk until intravascular signals could be observed. The Limberg score was recorded. A normal intestinal wall received a score of 0. Thickening of the intestinal wall without blood flow signal was assigned one point. Thickening of the intestinal wall with short blood vessels was assigned two points. Thickening of the intestinal wall with the presence of longer blood vessels received three points. Thickening of the intestinal wall and the presence of blood vessels connecting the mesentery received four points.

### SWE examination:

High frequency linear array probe was used to detect the thickest part of the intestinal wall. The grayscale image mode was switched to shear wave elastic ultrasound mode, and the imaging conditions were set to display the Young’s modulus value without pressure. The probe was placed perpendicular to the body surface; the patient was informed to hold their breath and the tissue elasticity image was viewed without pressure. The probe was left to stand for six seconds. The image was stabilized and framed for storage. The region of interest (ROI) was selected and placed axially at the 3/9 point position. The SD value of elastic modulus and the Young’s modulus value were recorded.

### Definition of endoscopic activity:

For children with CD, experienced endoscopists used the Simple Endoscopic Score for Crohn’s Disease (SES-CD; range 0-60) to assess endoscopic disease activity in the colon and terminal ileum. The intestine was divided into five parts (ileum, right colon, transverse colon, left colon, and rectum), and the endoscopic activity of each part was evaluated using four parameters: the presence and size of ulcers (score 0-3), the extent of ulcer surface (score 0-3), the area of affected surface (score 0-3), and the presence and degree of stenosis (score 0-3). The total score was then calculated, with scores of 0-2, 3-6, 7-15, and above 15 indicating remission, mild, moderate, and severe endoscopic activity, respectively. For children with ulcerative colitis, the Ulcerative Colitis Endoscopic Index of Severity (UCEIS) was used. UCEIS evaluates the affected intestinal segment from three aspects: vascular texture (0-2 points), bleeding condition (0-3 points), and erosion and ulceration (0-3 points). The total score of the most severe lesion was recorded as UCEIS, with a total score ranging from 0 to 8 points. UCEIS scores 0-1 for microscopic remission, 2-4 for mild, 5-6 for moderate, and 7-8 for severe endoscopic activity.

### Definition of poor prognosis:

For children with IBD, a 3-month follow-up period with recurrence or death was considered poor prognosis.

### Statistical analysis:

Data analysis was conducted according to a predefined statistical analysis plan using SPSS 21.0 software (IBM Corp, Armonk, NY, USA). Normal distribution measurement data were represented as mean ± standard deviation (SD), and a t-test was used to compare the IBD group and the non-IBD group. A repeated measures analysis of variance (ANOVA) was employed to compare children with varying degrees of IBD within the IBD group. The measurement data of skewed distribution was represented as median (interquartile range, IQR), and the rank sum test was used for comparison between the IBD group and the non-IBD group. The Kruskal-Wallis H test was used to examine the differences between children with varying degrees of IBD within the IBD group. Categorical variables were reported as frequency and percentage, and chi-square tests were used as appropriate to evaluate the differences between the IBD group and the non-IBD group.

The Spearman rank correlation coefficient (r) was used to calculate the correlation between biomarkers, ultrasound examination parameters, and the severity of IBD in children. P<0.05 was considered statistically significant. To find the optimal threshold for predicting poor future prognosis, a receiver operating characteristic (ROC) curve was constructed. The optimal cutoff point was determined by evaluating the sensitivity and specificity of various cutoff values. The accuracy of diagnostic testing was determined by the area under the ROC curve (AUC).

## RESULTS

This study included and analyzed 120 children with IBD and 120 non-IBD children. The entire cohort included 139 males and 101 females, with the age range of 2-12 years and a median age of 6 (5-8) years. In the IBD group, there were 67 cases of UC and 53 cases of CD. In terms of the disease severity, 31 children had mild, 51 moderate, and 38 severe disease. There were 25 cases with a poor prognosis, all of which were cases of recurrence, with no mortality. There was no significant difference in baseline data such as age, gender, and body mass index between the groups (P>0.05) (Table-I).

**Table-I:** Comparison of baseline data between two groups.

Baseline data	IBD group (n=120)	non-IBD group (n=120)	Z/χ_2_/t	P
Age (years)	6 (5-8)	7 (5-9)	-1.566	0.117
Gender, n (%)				
Male	74 (61.7)	65 (54.2)	1.385	0.239
Female	46 (38.3)	55 (45.8)		
Body Mass Index (kg/m2)	15.6 (13.05-17.8)	16.2 (13.5-17.9)	-0.798	0.425
Disease type, n (%)				
Ulcerative colitis	67 (55.8)	/	/	/
Crohn’s disease	53 (44.2)	/		
Degree of illness, n (%)				
Mild	31 (25.8)	/	/	/
Moderate	51 (42.5)	/		
Severe	38 (31.7)	/		
Poor prognosis	25 (20.8)			

IBD: inflammatory bowel disease.

The FC, FL, intestinal wall thickness, Limberg score, SD value, and Young’s modulus value of the IBD group were significantly higher than those of the non-IBD group (all P<0.05) (Table-II). The FC, FL, intestinal wall thickness, Limberg score, SD value, and Young’s modulus value were significantly higher in children with severe endoscopic activity compared to children with moderate endoscopic activity. Similarly, the values of these indexes were significantly higher in children with moderate endoscopic activity compared to those with mild endoscopic activity (P < 0.05) (Table-III).

**Table-II:** Comparison of fecal biomarkers and SWE examination parameters between two groups.

Variable	IBD group (n=120)	Non-IBD group (n=120)	t/Z	P
FC (ug/g)	153.11±54.28	27.99±8.93	24.917	<0.001
FL (ug/g)	78.87±29.8	4.94±1.24	27.157	<0.001
Intestinal wall thickness (mm)	5.22±1.27	2.57±0.76	19.593	<0.001
Limberg score (points)	3(2-3)	0(0-0)	-13.550	<0.001
SD value	4.79±1.23	0.58±0.2	37.066	<0.001
Young’s modulus value (kPa)	13.89±6.22	2.97±0.98	19.002	<0.001

IBD: inflammatory bowel disease. FC: fecal calprotectin. FL: fecal lactoferrin. SD: standard deviation.

**Table-III:** Comparison of fecal biomarkers and SWE examination results in children with different degrees of endoscopic activity.

Variable	Mild (n=31)	Moderate (n=51)	Severe (n=28)	F/H	P
FC (ug/g)	101.5±35.5	156.3±45.9	211.3±46.4	41.022	<0.001
FL (ug/g)	46.7±13.4	79.6±18.1	104.6±28.4	66.468	<0.001
Intestinal wall thickness (mm)	4.17±0.92	5.13±1.02	6.22±1.11	32.845	<0.001
Limberg score (points)	2 (1-2)	2 (2-3)	3 (2-3)	44.626	<0.001
SD value	3.69±0.81	4.73±1.04	5.85±0.97	41.8	<0.001
Young’s modulus value (kPa)	7.33±3.07	12.76±3.23	20.1±4.5	132.443	<0.001

FC: fecal calprotectin. FL: fecal lactoferrin. SD: standard deviation.

The Spearman test confirmed a significant positive correlation between FC, FL, intestinal wall thickness, Limberg score, SD value, and Young’s modulus value and the severity of IBD (P < 0.05) (Table-IV). The FC, FL, intestinal wall thickness, Limberg score, SD value, and Young’s modulus value of patients with poor prognosis in the IBD group were higher than those in children with good prognosis (all P<0.05) (Table-V).

**Table-IV:** Correlation analysis between fecal biomarkers and SWE examination parameters and endoscopic activity.

Item		FC	FL	Intestinal wall thickness	Limberg score	SD	Young’s modulus value
Endoscopic activity	r	0.703	0.737	0.603	0.598	0.657	0.819
	P	<0.001	<0.001	<0.001	<0.001	<0.001	<0.001

FC: fecal calprotectin. FL: fecal lactoferrin. SD: standard deviation.

**Table-V:** Comparison of fecal biomarkers and SWE examination results in children with different prognoses.

Variable	Good prognosis (n=95)	Poor prognosis (n=25)	t/Z	P
FC (ug/g)	141.1±50.6	229.8±37.2	8.186	<0.001
FL (ug/g)	69.4±23.7	115.8±23.7	8.716	<0.001
Intestinal wall thickness (mm)	4.89±1.11	6.51±1.07	6.521	<0.001
Limberg score (points)	2(2-3)	3(2-3)	-3.448	0.001
SD value	4.5±1.12	6.03±1	3.776	<0.001
Young’s modulus value (kPa)	11.7±4.7	21±4.9	8.693	<0.001

FC: fecal calprotectin. FL: fecal lactoferrin. SD: standard deviation.

**Fig.1:**
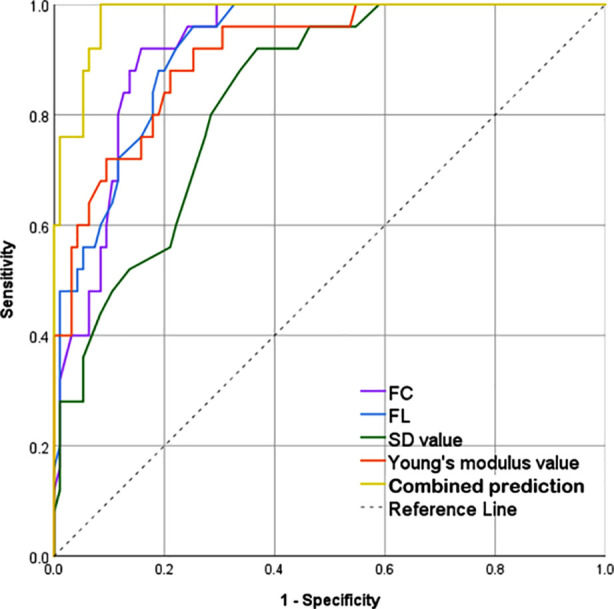
ROC curves. ***FC:*** fecal calprotectin. ***FL:*** fecal lactoferrin. ***SD:*** standard deviation.

The logistic regression model, using poor prognosis as the dependent variable and variables with statistically significant differences in Table-V as independent variables, identified FC, FL, SD values, and Young’s modulus as important risk factors for poor prognosis in children with IBD (P < 0.05; Table-VI). ROC curves to determine the optimal critical values for FC, FL, SD, and Young’s modulus are shown in Fig.1. The critical value of FC was 186 ug/g, with a sensitivity of 92%, specificity of 84.2%, and AUC of 0.921. The critical value of FL was 84.5 ug/g, with a sensitivity of 96%, specificity of 74.7%, and AUC of 0.920. The critical value of SD was 4.95, with a sensitivity of 92%, specificity of 63.2%, and AUC of 0.834. The critical value of Young’s modulus was 15.8 kPa, with a sensitivity of 88%, specificity of 78.9%, and AUC of 0.910. As shown in Table-VII, the combined predictive value of all four indicators was higher than that of each indicator (sensitivity of 100%, specificity of 91.6%, AUC of 0.983).

**Table-VI:** Analysis of risk factors associated with poor prognosis of inflammatory bowel disease.

Variable	β	S.E.	Waldχ^2^	P	OR	95%CI
FC	0.025	0.012	4.234	0.040	1.025	1.001-1.050
FL	0.051	0.024	4.417	0.036	1.053	1.003-1.104
SD value	1.153	0.585	3.883	0.049	3.169	1.006-9.979
Young’s modulus value	0.336	0.126	7.158	0.007	1.399	1.094-1.789

FC: fecal calprotectin. FL: fecal lactoferrin. SD: standard deviation.

**Table-VII:** The predictive value of fecal biomarkers and SWE examination parameters for poor prognosis of inflammatory bowel disease.

Index	Sensitivity	Specificity	Optimal Cutoff	AUC of ROC	P
FC	92%	84.2%	186 (ug/g)	0.921	0.024
FL	96%	74.7%	84.5 (ug/g)	0.920	0.025
SD value	92%	63.2%	4.95	0.834	0.039
Young’s modulus value	88%	78.9%	15.8 (kPa)	0.910	0.030
Combined prediction	100%	91.6%		0.983	0.009

FC: fecal calprotectin. FL: fecal lactoferrin. SD: standard deviation.

## DISCUSSION

This study aimed to investigate the value of SWE ultrasound parameters combined with fecal biomarker testing in the diagnosis and treatment evaluation of children with IBD. The results showed that the diagnosis of IBD was associated with higher FC, FL, intestinal wall thickness, Limberg score, SD value, and Young’s modulus value.

This study indicate that FC, FL, intestinal wall thickness, Limberg score, SD value, and Young’s modulus value allow to effectively distinguish children with IBD from non-IBD children, which is consistent with the research results of Ma et al.^12^ and Singh et al.^13^ FC and FL are fecal biomarkers of intestinal inflammation, and their elevation in children with IBD reflects the inflammatory state of the intestine.^10^-^12^,^14^ The intestinal wall thickness, Limberg score, SD value, and Young’s modulus value were detected by SWE.^13^,^15^ The increase in intestinal wall thickness caused by IBD-associated inflammation leads to tissue proliferation, edema, and other changes, while the increase in Limberg score, SD value, and Young’s modulus value is related to changes in the elasticity of the intestinal wall.^16^ Inflammation can disrupt the normal structure and function of the intestinal wall, resulting in a decrease in its elasticity, which differs from that of non-IBD children.^13^,^15^,^17^

The results of this study found that FC, FL, intestinal wall thickness, Limberg score, SD value, and Young’s modulus value are positively correlated with the severity of endoscopic activity of IBD. It is plausible that the intestinal wall of children with severe endoscopic activity may thicken more significantly due to long-term inflammatory stimulation, leading to higher levels of FC and FL and poorer elasticity, as indicated by the changes in the Limberg score, SD value, and Young’s modulus value.^9^-^13^ This correlation provides a basis for clinicians to assess the degree of endoscopic activity by using these indicators.^12^-^15^,^17^ Ding et al.^18^ showed that intestinal wall thickness and elastic modulus may change in patients with IBD. Therefore, IBD-related changes in intestinal stiffness that are detected by the SWE technology may serve as a potentially important biomarker for predicting intestinal stenosis in children with IBD.^17^,^18^

The logistic analysis results indicated that high FC, FL, SD values, and Young’s modulus values are all risk factors for poor prognosis in children with IBD. Therefore, the above indicators can not only be used for disease diagnosis and assessment but also have important significance in predicting the prognosis of pediatric patients. On diagnosis, IBD patients who present with very high FC and FL values, significantly thickened intestinal walls, and poor elasticity index have a poorer prognosis.^19^,^20^ Therefore, by assessing these indexes, clinicians can develop more proactive treatment plans. The ROC curve results also indicated that FC, FL, SD values, and Young’s modulus values have high predictive values for poor prognosis in IBD patients, and the combined prediction value of the four indicators is significantly higher than that of each predictor alone.

SWE is an accurate, non-invasive biomarker for assessing histological disease activity in IBD, and its accuracy is further enhanced when combined with FC and FL.^20^-^23^ In the current study, we have confirmed that SWE combined with FC and FL has higher value in the diagnosis and assessment of IBD. This combined approach can reduce the need for colonoscopy by supporting accurate bedside decision-making in children with IBD.

### Limitations

First of all, this is a single-center retrospective analysis that included non-randomized enrollment of pediatric patients; thus, the results may not be generalizable to other settings due to limitations in sample diversity. Second, the follow-up period was only three months. Therefore, the statistics on the incidence of poor prognosis are not comprehensive. Third, despite centralized and standardized training and practices to minimize variability in data acquisition by different operators, SWE indicators can still be affected by human or technical factors. Further prospective studies with large sample sizes are needed to validate the conclusions of this study.

## CONCLUSION

SWE parameters are accurate, non-invasive biomarkers of histological disease activity in IBD, and their accuracy is further enhanced when combined with FC and FL. Combining SWE and fecal biomarker testing has high application value in diagnostics, assessing the degree of endoscopic activity, and prognosticating patients with IBD.

### Authors’ contributions:

**LY:** Literature search, Study design and manuscript writing.

**BL, HH, QZ, Qian Zhang and ZZ:** Data collection, data analysis and interpretation. Critical review.

**LY:** Manuscript revision, validation and is responsible for the integrity of the study.

All authors have read and approved the final manuscript.
